# Inhibition of TACE Activity Enhances the Susceptibility of Myeloma Cells to TRAIL

**DOI:** 10.1371/journal.pone.0031594

**Published:** 2012-02-28

**Authors:** Kumiko Kagawa, Ayako Nakano, Hirokazu Miki, Asuka Oda, Hiroe Amou, Kyoko Takeuchi, Shingen Nakamura, Takeshi Harada, Shiro Fujii, Kenichiro Yata, Shuji Ozaki, Toshio Matsumoto, Masahiro Abe

**Affiliations:** 1 Department of Medicine and Bioregulatory Sciences, University of Tokushima Graduate School of Medicine, Tokushima, Japan; 2 Division of Internal Medicine, Tokushima Prefectural Central Hospital, Tokushima, Japan; University of Nebraska – Lincoln, United States of America

## Abstract

**Background:**

TNF-related apoptosis-inducing ligand/Apo2 ligand (TRAIL/Apo2L) selectively induces apoptosis in various cancer cells including myeloma (MM) cells. However, the susceptibility of MM cells to TRAIL is largely low in most of MM cells by yet largely unknown mechanisms. Because TNF-α converting enzyme (TACE) can cleave some TNF receptor family members, in the present study we explored the roles of proteolytic modulation by TACE in TRAIL receptor expression and TRAIL-mediated cytotoxicity in MM cells.

**Methodology/Principal Findings:**

MM cells preferentially expressed death receptor 4 (DR4) but not DR5 on their surface along with TACE. Conditioned media from RPMI8226 and U266 cells contained a soluble form of DR4. The DR4 levels in these conditioned media were reduced by TACE inhibition by the TACE inhibitor TAPI-0 as well as TACE siRNA. Conversely, the TACE inhibition restored surface levels of DR4 but not DR5 in these cells without affecting DR4 mRNA levels. The TACE inhibition was able to restore cell surface DR4 expression in MM cells even in the presence of bone marrow stromal cells or osteoclasts, and enhanced the cytotoxic effects of recombinant TRAIL and an agonistic antibody against DR4 on MM cells.

**Conclusions/Significance:**

These results demonstrate that MM cells post-translationally down-modulate the cell surface expression of DR4 through ectodomain shedding by endogenous TACE, and that TACE inhibition is able to restore cell surface DR4 levels and the susceptibility of MM cells to TRAIL or an agonistic antibody against DR4. Thus, TACE may protect MM cells from TRAIL-mediated death through down-modulation of cell-surface DR4. It can be envisaged that TACE inhibition augments clinical efficacy of TRAIL-based immunotherapy against MM, which eventually becomes resistant to the present therapeutic modalities.

## Introduction

Multiple myeloma (MM) remains essentially incurable for the vast majority of patients by conventional anti-tumor therapies, which has led to increased interest in clinical application of various forms of immune therapies. One such approach is the application of TNF-related apoptosis-inducing ligand/Apo2 ligand (TRAIL) or TRAIL agonistic antibodies [1]–[4]. Because TRAIL is not cytotoxic to normal tissues unlike Fas ligand and TNF-α, TRAIL-mediated immunotherapy is tumor-specific, and regarded as an attractive maneuver against various cancers including MM [5], [6], [7]. However, the susceptibility of MM cells to TRAIL has been demonstrated to be largely low in most of MM cells, which limits clinical applications of TRAIL-mediated immunotherapy. Therefore, the development of novel therapeutic strategies to vitalize TRAIL-induced apoptotic signaling in MM cells remains an important clinical issue.

TRAIL binds to 2 different proapoptotic receptors, death receptor 4 (DR4) and DR5. TRAIL and its receptors belong to TNF-like ligand/receptor family members. TNF-α converting enzyme (TACE) is known as a sheddase for TNF-like ligands/receptors to modulate the biological activities of some of these family members such as TNF-α [8], [9]. Enforced expression of tissue inhibitor of metalloproteinases-3 (TIMP-3), an endogenous inhibitor for TACE, has been reported to up-modulate surface levels of some TNF receptor family members including DR4 and Fas in metastatic melanoma cell lines [10]. However, the role of TACE in surface expression of TNF-like ligand/receptor family members and the inhibition of TACE activity in TRAIL-mediated cytotoxicity against MM cells has not been studied. In the present study, we therefore investigated the role for TACE in TRAIL and its receptor editing on MM cells as well as the effect of TACE inhibition on TRAIL-triggered cytotoxicity in MM cells. We demonstrate herein that MM cells post-translationally down-modulate the cell surface expression of the TRAIL receptor DR4 through ectodomain shedding by endogenous TACE, and that TACE inhibition is able to restore cell surface DR4 expression and the susceptibility of MM cells to TRAIL or an agonistic antibody against DR4.

## Results

### Most hematopoietic malignant cells expresses TACE but represses TIMP-3

**Figure 1:**
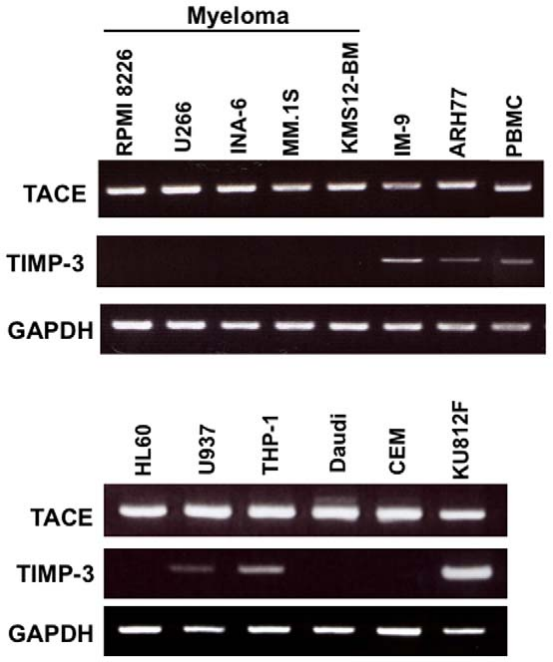
The expression of TACE and TIMP-3 in various types of hematopietic malignant cells. TACE and TIMP-3 mRNA expression was analysed by RT-PCR in MM, B-lymphoid and leukemic cell lines as indicated as well as normal PBMCs. Human GAPDH was used as a housekeeping gene for quantity normalization.

Surface levels of some TNF receptor family members have been suggested to be affected by enforced expression of TIMP-3 in metastatic melanoma cell lines [10]. However, the expression of TACE and its endogenous inhibitor, TIMP-3, has not been precisely studied in malignant hematopoietic cells. Therefore, we first investigated the expression of TACE and TIMP-3 in various types of malignant cells. TACE mRNA was constitutively expressed in all cell lines tested including RPMI8226, U266, INA-6, MM.1S AND KMS12 MM cell lines, HL-60, U937 leukemic cell lines, and EBV-transformed B cell lines as well as normal PBMCs (Figure 1). However, these MM cell lines and most of leukemic cells showed only marginal expression of TIMP-3 mRNA, while the B cell lines and normal PBMCs constitutively expressed TIMP-3. These results suggest that TACE activity is enhanced along with repression of TIMP-3 expression in most of hematological malignant cells including MM and leukemic cells.

### Cell surface DR4 levels are down-modulated by TACE in MM cells

**Figure 2:**
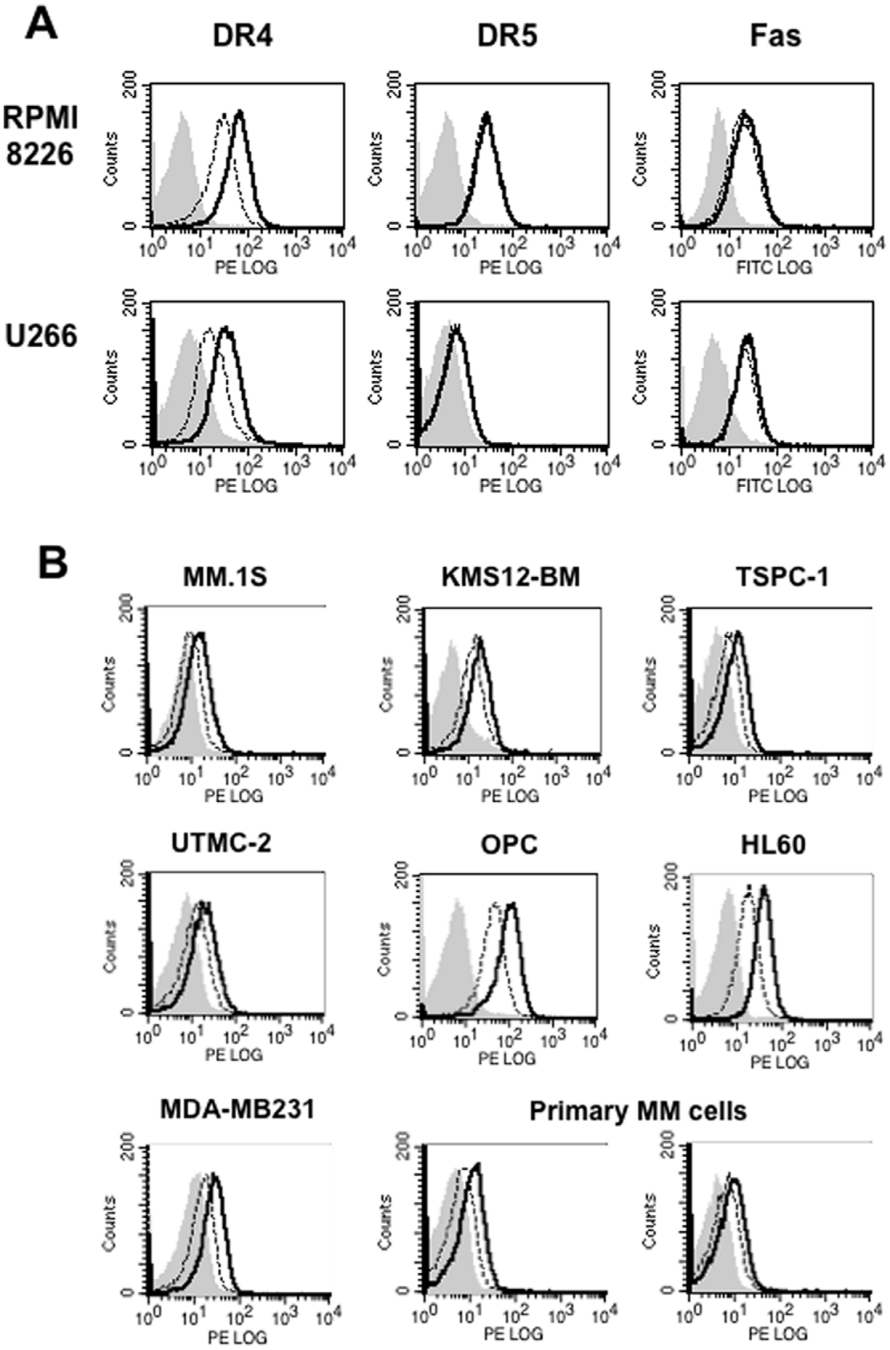
Up-modulation of cell surface DR4 levels by TACE inhibition. **A.** RPMI8226 and U266 cells were cultured for 24 hours in the absence (dotted lines) or presence (solid lines) of TAPI-0, a TACE inhibitor, at 10 µM. Surface expression of DR4, DR5 and Fas was analyzed by flow cytometry. **B.** Surface expression of DR4 was analyzed by flow cytometry in various types of cell lines as indicated as well as primary MM cells after culturing for 24 hours in the absence (dotted lines) or presence (solid lines) of TAPI-0 at 10 µM. Background staining with normal IgG is shown in gray.

In order to clarify the role of TACE, we next determined whether TACE affects the surface levels of TNF-like ligand/receptor family members on MM cells. Both RPMI8226 and U266 cells constitutively expressed DR4 and Fas on their surface as determined by flow cytometry (Figure 2A). DR5 was expressed in RPMI8226 cells but marginally in U266. To clarify the role of TACE in editing of these molecules on MM cells, we examined their surface levels upon TACE inhibition by the TACE inhibitor TAPI-0. After incubating with TAPI-0 for 24 hours the surface levels of DR4 were enhanced on both RPMI8226 and U266 cells, while DR5 and Fas showed no appreciable change (Figure 2A). Up-modulation of surface DR4 levels by the TAPI-0 treatment were also obtained in other MM cell lines, MM.1S, KMS12, TSPC-1, UTMC-2 and OPC, primary MM cells, leukemic cell lines, HL-60, and MD-MB-231 breast cancer cell line (Figure 2B). These results suggest that surface DR4 expression is down-modulated by endogenous TACE in malignant tumors including MM.

### TACE mediates the ectodomain shedding of DR4 in MM cells

**Figure 3:**
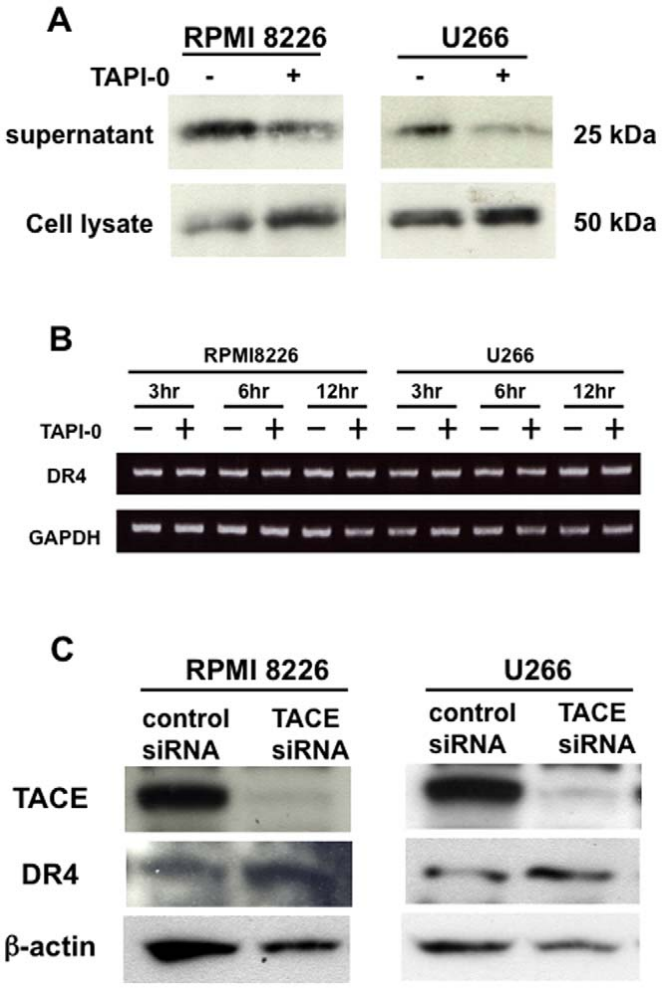
TACE is responsible for ectodomain shedding of DR4. **A.** Cell lysates and culture supernatants were harvested after culturing RPMI8226 and U266 cells for 48 hours in the absence or presence of TAPI-0 at 10 µM. DR4 immunoreactivity was analyzed by immunobloting with an antibody against an DR4 extracellular domain. **B.** RPMI8226 and U266 cells were cultured for different hours as indicated in the absence or presence of TAPI-0 at 10 µM. DR4 mRNA expression was analysed by RT-PCR in RPMI8226 and U266 cells. GAPDH was used for quantity normalisation. **C.** RPMI8226 and U266 cells seeded in 24-well plates were transfected with TACE or control scrambled siRNA. After culturing for 48 hours, cell lysates were harvested. DR4 immunoreactivity was analysed by immunobloting. β-actin was used as a loading control.

To clarify ectodomain shedding of DR4 in MM cells by TACE, we next examined the effects of TACE inhibition on the levels of DR4 in both cell lysates and conditioned media by Western blotting using antibody against DR4 ectodomain. Conditioned media from RPMI8226 and U266 cells contained an immunoreactive DR4 protein with a molecular size smaller than that observed in their cell lysates. Interestingly, the treatment with TAPI-0 reduced DR4 levels in culture supernatants of these MM cells, while increasing them in their cell lysates (Figure 3A). However, the TAPI-0 treatment showed no significant effects on DR4 mRNA expression in RPMI8226 and U266 cells at 3, 6 and 12 hours (Figure 3B). We further examined the DR4 mRNA levels in different MM cell lines, including RPMI8226, U266, OPC, TSPC-1, KMS12-BM, and MM.1S cells, at 6, 12 and 48 hours in the presence or absence of TAPI-0. The DR4 mRNA levels showed no appreciable change in the presence of TAPI-0 in these cells at 48 hours as well as 6 and 12 hours (Figure S1), although surface protein levels of DR4 were up-modulated by the TAPI-0 treatment in these cells (Figures 2A and 2B). From these results, the up-modulation of surface DR4 levels by TAPI-0 is suggested to be largely due to the suppression of its ectodomain shedding. To further confirm the role of TACE in ectodomain shedding of DR4, we examined the effects of TACE siRNA on surface expression of DR4. Treatment with TACE siRNA suppressed TACE expression at a protein level in RPMI8226 and U266 cells in parallel with resumption of DR4 levels in their cell lysates (Figure 3C). These observations are consistent with the notion that surface DR4 levels are post-translationally down-modulated by endogenous TACE in MM cells.

### TACE inhibition enhances the susceptibility of MM cells to TRAIL or an agonistic antibody against DR4

**Figure 4:**
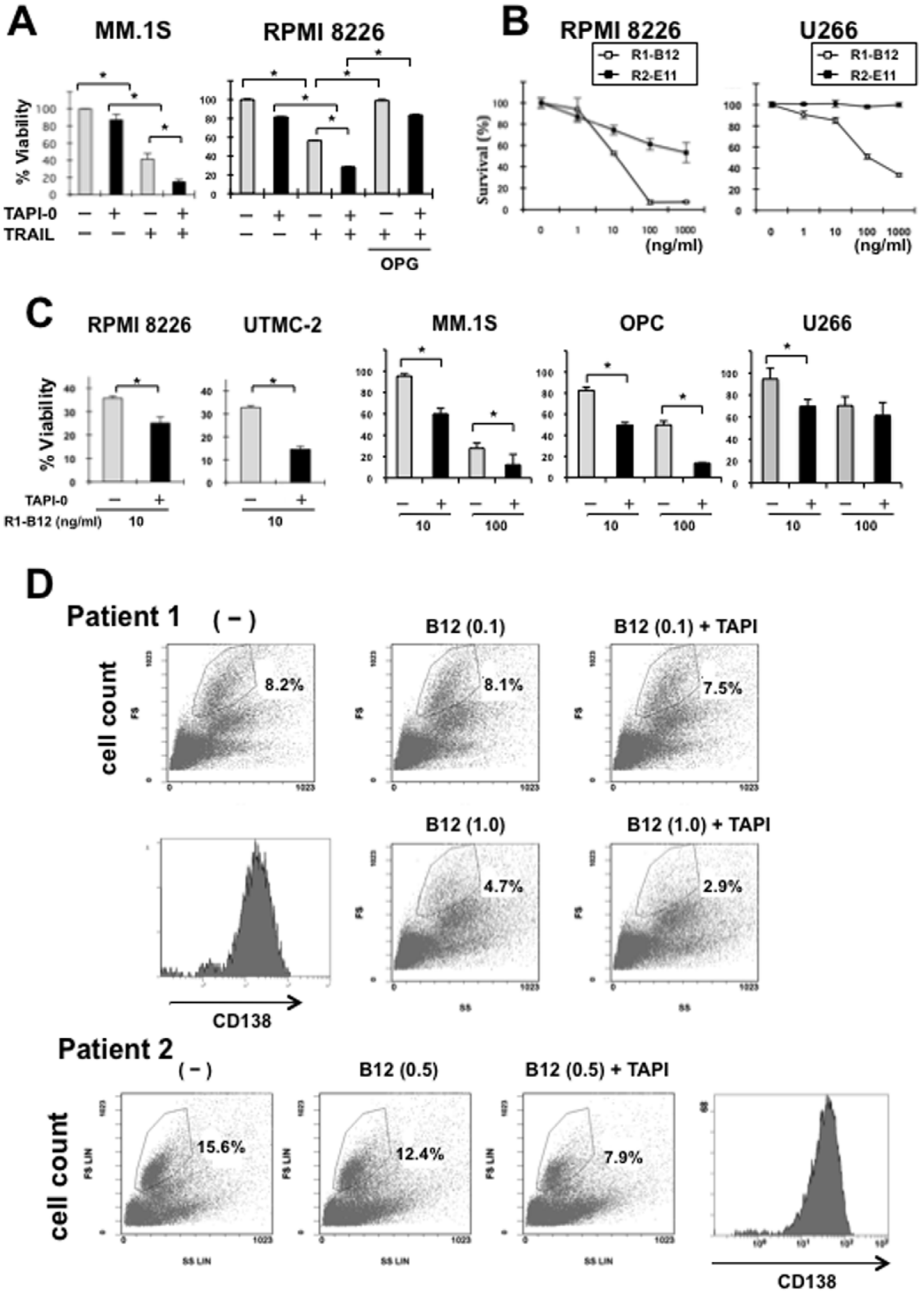
TACE inhibition enhances the cytotoxic effects of TRAIL and R1-B12 on MM cells. **A.** MM.1S and RPMI8226 cells were cultured in quadruplicate in 24-well plates in the absence or presence of recombinant TRAIL at 20 ng/ml. TAPI-0 and rh osteoprotegerin (OPG) was added at 10 µM and 1 µg/ml, respectively, as indicated. **B.** RPMI8226 and U266 cells were cultured in quadruplicate in 24-well plates in the absence or presence of the anti-DR4 agonistic antibody R1-B12 (□) or the anti-DR5 agonistic antibody R2-E11 (▪) at the different concentrations as indicated. **C.** RPMI8226, UTMC-2, MM.1S and OPC cells were cultured in quadruplicate in 24-well plates in the absence or presence of R1-B12. R1-B12 was added at the different concentrations as indicated. TAPI-0 was added at 10 µM. After culturing for 24 hours, cell viability was determined by WST-8 assay. The results are expressed as % change from controls without the treatment (mean +/− SD). *, p<0.05. **D.** Per cent distribution of CD138-positive MM cells. MM cell fractions were gated according to their expression of CD138, a specific marker for MM cells, as marked in the histograms. X and Y axis represents a side scatter (SS) and forward scatter (FS), respectively. CD138 staining within the gated cells in the control (left lower) indicates that MM cell fraction was correctly gated. Per cent distribution of CD138-positive MM cells was counted within whole bone marrow cells in in the histograms.

We next investigated whether restoration of cell-surface DR4 levels by TACE inhibition is able to enhance the cytotoxic effects of TRAIL or an agonistic antibody against DR4 on MM cells. Treatment with recombinant TRAIL reduced the viability of MM.1S and RPMI8226 cells (Figure 4A). Combination with the TACE inhibitor TAPI-0 further reduced the viability of these cells, although TAPI-0 alone did not show significant effects. Addition of rh osteoprotegerin, an inhibitory decoy receptor for TRAIL, mostly abolished the cytotoxic effects of recombinant TRAIL on RPMI8226 cells in both the absence and presence of TAPI-0 (Figure 4A), which indicates that the TAPI-0's effects are TRAIL-dependent. Besides recombinant TRAIL, R1-B12, an anti-DR4 agonistic antibody, induced cell death in DR4-expressing RPMI8226 and U266 cells (Figure 4B). However, the anti-DR5 agonistic antibody R2-E11 induced cell death only in RPMI8226 cells but not in U266 cells at relatively higher concentrations, which is consistent with surface expression levels of DR5 on these cells. TACE inhibition by TAPI-0 treatment enhanced the cytotoxic effects of R1-B12 on RPMI8226, UTMC-2, MM.1S and OPC cells (Figure 4C). Although MM.1S cells appeared to be susceptible to TRAIL in our experimental condition, their susceptibility to TRAIL has been differently reported [11]–[13].

To substantiate the laboratory observations, we examined the cytotoxic effects of R1-B12 on bone marrow cells from 2 patients with MM in the presence or absence of the TACE inhibitor TAPI-0. MM cell fractions were gated according to their expression of CD138, a specific marker for MM cells, as marked in the histograms in flow cytometry (Figure 4D). Per cent distribution of CD138-positive MM cells in whole bone marrow cells was reduced by the treatment with R1-B12 at 1.0 µg/ml in patient 1 and at 0.5 µg/ml in patient 2. The combinatory treatment with TAPI-0 further reduced the % distribution of CD138^+^ MM cells (4.7% vs. 2.9% in patient 1 and 12.4% vs. 7.9% in patient 2, respectively). These results suggest that TACE inhibition is able to sensitize primary MM cells to TRAIL-mediated cell death, although higher doses of R1-B12 were required to induce cell death in primary MM cells compared to MM cell lines.

Although U266 cells express DR4 on their surface (Figure 2A), they appear to be rather resistant to agonistic R1-B12 compared to RPMI8226 cells (Figure 4B). Treatment with TAPI-0 slightly potentiated the cytotoxic effects of R1-B12 at 10 nM but not at 100 nM on U266 cells (Figure 4C). Thus, U266 cells appear to remain resistant to R1-B12 even after up-modulation of DR4 with the TACE inhibition. Besides the surface levels of DR, the DR-mediated pro-apoptotic signaling is intracellularly regulated. C-FLICE-like interleukin protein (c-FLIP) is a predominant negative regulator for the DR-mediated intracellular pro-apoptotic signaling, which inhibits caspase8 activation. U266 cells were found to constitutively over-express c-FLIP; and the knockdown of c-FLIP by its siRNA significantly restored the cytotoxic effects of R1-B12 on U266 cells (manuscript in preparation). Therefore, inhibition of the DR-mediated pro-apoptotic signaling pathway by c-FLIP is suggested to largely contribute to the resistance of U266 cells to R1-B12. Both the up-modulation of surface DR4 by TACE inhibition and the potentiation of the DR-mediated pro-apoptotic signaling are suggested to be required for full restoration of the susceptibility of MM cells to TRAIL-mediated immunotherapy. The osteosarcoma cell line MG63 was found to express no appreciable DR4 protein on their surface but weakly DR4 mRNA. In this cell line, treatment with TAPI-0 showed no appreciable change in the surface levels of DR4 and did not induce the cytotoxic effects of R1-B12 (data not shown). PBMCs from normal subjects and CD138-negative normal hematopoietic cells in the bone marrow also showed no detectable levels of surface DR4 in flow cytometry. The treatment with TAPI-0 induced neither surface DR4 nor cell death by R1-B12 in these normal cells. Therefore, the TACE inhibition is suggested to be able to effectively enhance surface DR4 levels and R1-B12-mediated cell death in malignant cells expressing at detectable levels of surface DR4. Collectively, the present strategy with R1-B12 in combination with TACE inhibition can work well for malignant cells exhibiting detectable levels of DR4 without attenuation of the DR-mediated intracellular pro-apoptotic signaling.

**Figure 5:**
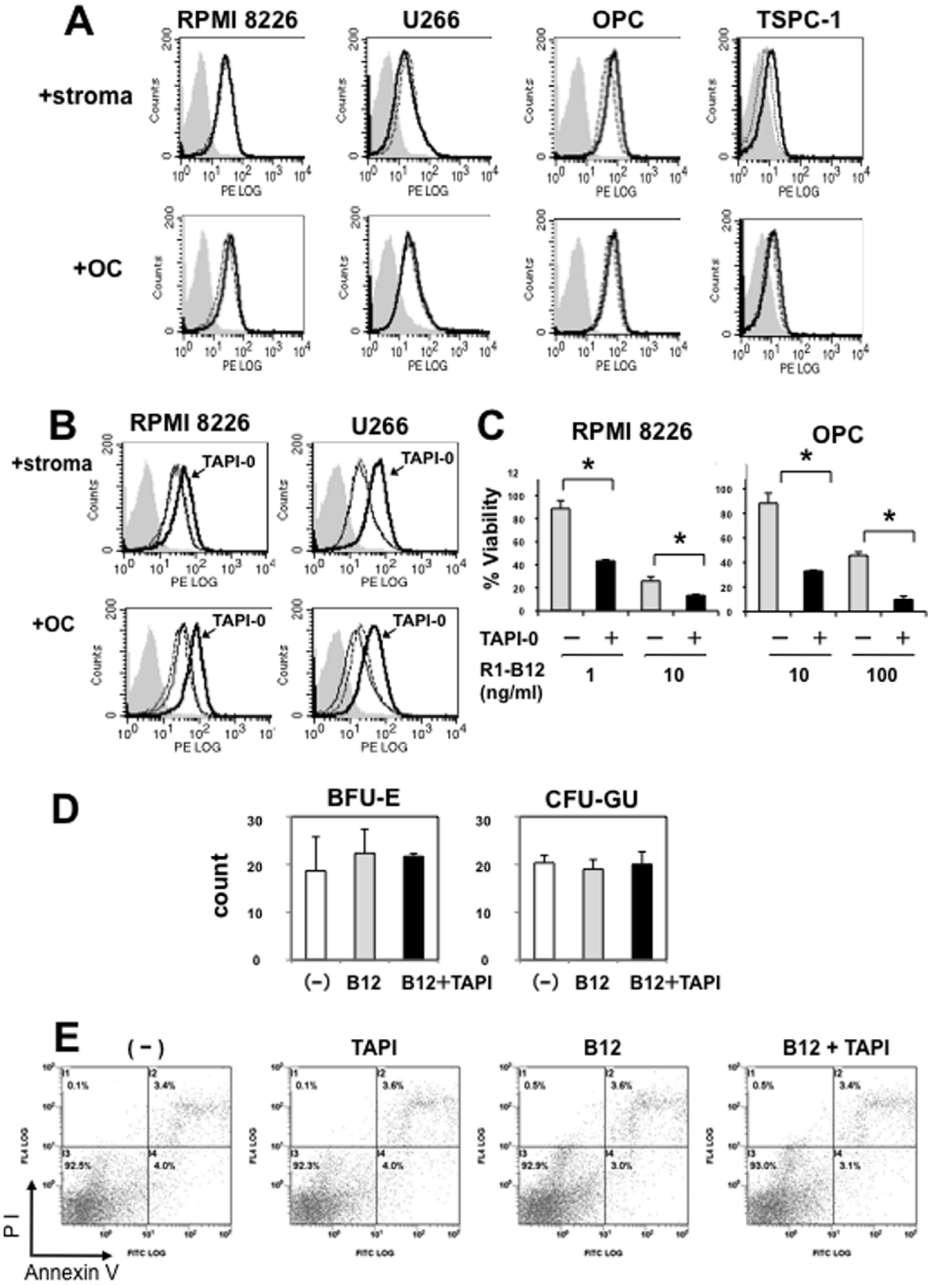
TACE inhibition enhances anti-MM effects of R1-B12 in the presence of bone marrow stromal cells. **A.** Surface DR4 levels on MM cells in cocultures with bone marrow stromal cells or osteoclasts. MM cell lines as indicated were cultured alone (dotted line) or cocultured (solid line) for 2 days with bone marrow stromal cells or osteoclasts (OCs). **B.** The effects of TAPI-0 on surface DR4 levels on MM cells in the cocultures. MM cell lines as indicated were cultured alone (solid line) or cocultured (dotted line) with bone marrow stromal cells or osteoclasts (OCs). TAPI-0 was added at 10 µM into the cocultures as indicated. After culturing for 2 days, MM cells were harvested, and surface expression of DR4 was analyzed by flow cytometry. **C.** RPMI8226 and OPC cells were cocultured with bone marrow stromal cells. The anti-DR4 agonistic antibody R1-B12 at the different concentrations and TAPI-0 at 10 µM were added as indicated. After culturing for 2 days, the viability of was RPMI8226 cells analysed by WST-8 assay. Data are expressed as the change in viable cell numbers relative to a control without any agents (mean +/− SD). *, p<0.05. **D.** Colony forming cell assays. CD138-negative bone marrow cells were cultured in the absence or presence of R1-B12 or R1-B12 plus TAPI-0 for 14 days, and BFU-E and CFU-GM were counted. **E.** The effects of R1-B12 plus TAPI-0 on normal hematopoietic cells. CD138-negative normal hematopoietic cells were isolated from the bone marrow sample from a patient with MM, and treated for 2 days with R1-B12 or TAPI-0 alone, or both in combination. The cells were then subject to annexin V-FITC and PI dual staining.

Because bone marrow stromal cells or osteoclasts confer resistance against apoptosis in MM cells, we next asked whether the TACE inhibition is able to enhance the DR4-mediated cytotoxic effects on MM cells in the presence of bone marrow stromal cells or osteoclasts. Surface DR4 levels seemed marginally or only slightly affected in MM cells including RPMI8226, U266, OPC and TSPC-1cells after coculturing with bone marrow stromal cells or osteoclasts (Figure 5A). Similar to MM cells cultured alone, TAPI-0 treatment increased surface DR4 levels in MM cells even in the presence of bone marrow stromal cells (Figure 5B). The anti-DR4 agonistic antibody R1-B12 was able to induce cell death in MM cells in the presence of bone marrow stromal cells (Figure 5C), although bone marrow stromal cells are known to secrete a large amount of osteoprotegerin, a TRAIL inhibitor [14], [15]; TACE inhibition by TAPI-0 further enhanced the DR4-mediated cell death with the antibody (Figure 5C). Therefore, these results collectively suggest that restoration of DR4 by TACE inhibition enhances the susceptibility of MM cells to DR4-mediated cell death.

The effects of our strategy on normal hematopoietic cells is an important issue. To address this, we conducted hematopoietic progenitor assays upon the treatment using R1-B12 with or without TAPI-0. Addition of R1-B12 or R1-B12 plus TAPI-0 showed no effects on the formation of CFU-GM and BFU-E from CD138-negative normal bone marrow hematopoietic cells (Figure 5D). Moreover, we examined the cytotoxic effects of our strategy on normal bone marrow hematopoietic cells. The treatment with R1-B12 or TAPI-0 alone, or both in combination showed no appreciable change in the induction of apoptotic nor propidium iodine (PI)-positive dead cells in CD138-negative normal bone marrow hematopoietic cells isolated from a patient with MM (Figure 5E). Therefore, our strategy appears not to sensitize normal hematopoietic cells to TRAIL-mediated cell death. Furthermore, CD34^+^ hematopoietic progenitor cells purified from human cord blood, adult peripheral blood or adult bone marrow have been demonstrated not to express detectable levels of surface TRAIL receptors and be resistant to TRAIL [16]. More recently, Mizrahi K, et al. reported that TRAIL does not trigger apoptosis in hematopoietic progenitors and rather enrich myeloid progenitors [17]. Therefore, our strategy appears not to sensitize normal hematopoietic cells to TRAIL-mediated cell death.

## Discussion

Cytokine and growth factor signaling is controlled by the amount of relevant ligands as well as the expression levels of their cognate receptors. TACE cleaves and releases various cell surface proteins from the plasma membrane, including cytokines and cytokine receptors [8], [9], [18]–[20]. The cleavage of cytokine receptors results in loss of their ligand binding capacity and shedding of their soluble forms as a decoy receptor to interrupt binding of their ligands to them, thereby drastically disrupting the activity of cytokines as well as signal transduction through cytokine receptors [18]. The present study demonstrates that DR4 on the surface of tumor cells with enhanced TACE activity is post-translationally down-modulated through TACE-mediated shedding. TACE-mediated shedding appears to be an important mechanism for the reduction of surface DR4 levels on MM cells, which may blunt TRAIL-mediated apoptosis by surrounding immune cells expressing TRAIL to protect MM cells. Up-modulation of surface DR4 levels by inhibiting TACE activity is able to restore the susceptibility of MM cells to TRAIL.

Although bone marrow stromal cells and osteoclasts create a microenvironment which confers resistance against apoptosis in MM cells, TACE inhibition is able to restore cell surface DR4 expression in MM cells even in the presence of bone marrow stromal cells or osteoclasts and enhance the cytotoxic effects of an agonistic antibody against DR4 on MM cells (Figures 5B and 5C). Because tumor immunity is suppressed in MM, the efficacy of immunotherapies utilizing host immune cells is largely limited in patients with MM [21]. In addition, osteoprotegerin, an endogenous inhibitor for TRAIL, is abundantly secreted by bone marrow stromal cells in the bone marrow in which MM cells preferentially reside [15]. Because an agonistic antibody against DR4 can act on MM cells without host immune cells and in the presence of osteoprotegerin, the anti-DR4 agonistic antibody with TACE inhibitors may become a novel immunotherapeutic approach against MM.

TNF-α is regarded as an important cytokine produced by MM cells and surrounding cells in the bone marrow in MM [22]. TNF-α is activated from inactive pro- TNF-α by TACE, and released from a cell surface. It directly stimulates MM cell growth and survival as well as creates pathological microenvironment with enhanced ostesclastogenesis and angiogeneisis [23]–[25]. TNF-α activates NF-κB in MM cells, which leads to enhance the expression of c-FLIP, a potent inhibitor for caspase8 activation, to attenuate the death receptor-mediated apoptotic pathway [26], [27]. Therefore, the suppression of TNF-α activation by TACE inhibition may also help TRAIL-induced MM cell death.

Collectively, it can be envisaged that TACE inhibition augments clinical efficacy of TRAIL-based immunotherapy against MM which eventually becomes resistant to the present therapeutic modalities. This novel therapeutic modality can also be applied to other types of cancers exerting TACE activity. However, malignant cell resistance to TRAIL is largely caused by the down-regulation of TRAIL receptor expression and the suppression of its downstream pro-apoptotic signaling. To resume the sensitivity to TRAIL, the development of novel therapeutic maneuvers is required to up-modulate surface TRAIL receptors along with the implementation of stimulators of the DR-mediated pro-apoptotic signaling. The combination of TACE inhibition with activators for caspase8 such as histon deacetylase inhibitors, lenalidomide or bortezomib may further improve the efficacy of TRAIL-based immunotherapy. Overall clinical benefits of these strategies will be determined in well-deigned clinical studies.

## Materials and Methods

### Reagents

The following reagents were purchased from the indicated manufacturers: a TACE inhibitor, TAPI-0, from Calbiochem (Bad Soden, Germany); recombinant human (rh) TRAIL, osteoprotegerin, mouse monoclonal antibodies against human TRAIL, DR4, DR5, decoy receptors for TRAIL, DcR1 and DcR2, Fas ligand, Fas, CD40 and CD27 from R&D Systems (Minneapolis, MN); rabbit polyclonal anti-human DR4 (ectodomain-specific) antibody from Santa Cruz Biotechnology (Santa Cruz, CA); fluorescein isothiocyanate (FITC)-conjugated goat anti-mouse IgG from BD Pharmingen (San Diego, CA); rabbit anti-β-actin from Sigma (St. Louis, MO); horseradish peroxidase (HRP)- anti-rabbit IgG from Cell Signaling Technology (Beverly, MA); and goat F(ab′)_2_ anti-human IgG Fc from Pierce (Rockford, IL). The human monoclonal anti-DR4 agonistic antibody R1-B12 and the anti-DR5 agonistic antibody R2-E11 were kindly provided by Kyowa Hakko Kirin Co. Ltd. (Tokyo, Japan).

### Cells and cultures

Human MM cell lines, RPMI8226 and U266, human myeloid cell lines, HL-60, KG1a, KU812F and U937, human lymphoid cell lines, ARH77 and IM-9, and human non-hematopoietic cell lines, MD-MB231, MCF7 and Colo205, were obtained from American Type Culture Collection (ATCC) (Rockville, MD). The MM cell line INA6, MM.1S, UTMC-2 and KMS12-BM was kindly provided by Dr. Renate Burger (University of Kiel, Kiel, Germany), Dr. Steven Rosen (Northwestern University, Chicago, IL), Dr. Alan Solomon (University of Tennessee, TN) and Dr. Otsuki (Kawasaki Medical School, Okayama, Japan), respectively. TSPC-1 and OPC MM cell lines were established in our laboratory [28]. Bone marrow mononuclear cells were isolated by Ficoll-Hypaque density gradient centrifugation (Pharmacia LKB Biotechnology, Uppsala, Sweden) from heparinized bone marrow blood drawn from patients with MM. MM cells were further purified from bone marrow mononuclear cells with positive selection using CD138 (Syndecan-1) microbeads and Miltenyi magnetic cell sorting system (Miltenyi Biotec, Auburn, CA) according to the manufacture's instruction. The purity of the MM cells was greater than 95%. Primary stromal cells derived from fresh BM aspirates were isolated and cultured as we previously described [28], [29]. These cells are a homogeneous population of spindle-shaped cells expressing CD44, CD73, CD90 and CD105, but neither CD45 nor factor VIII. OCs were generated from peripheral blood mononuclear cells (PBMCs) as previously reported [15]. Cells were cultured in αMEM supplemented with 10% fetal bovine serum (FBS), 2 mM of L-glutamine (Sigma), 100 U/ml of penicillin G and 100 mg/ml of streptomycin (Sigma). All procedures involving human specimens were performed with written informed consent according to the Declaration of Helsinki and using a protocol approved by the Institutional Review Board for human protection.

### Flow cytometry

Cell preparation and staining for flow cytometry were performed as described previously [29]. Approximately 10^6^ cells were incubated in 100 µl PBS with 2% human γ-globulin with saturating concentrations of different FITC-conjugated monoclonal antibodies on ice for 40 minutes and the washed. Samples were analyzed by flow cytometry using EPICS-Profile (Coulter Electronics, Hialeah, FL).

### Western blot analysis

Cells were collected and lysed in lysis buffer (Cell Signaling) supplemented with 1 mM phenylmethylsulfonyl fluoride and protease inhibitor cocktail solution (Sigma). Cell lysates and conditioned media were electrophoresed in 10% SDS-PAGE gel and blotted onto polyvinylidene difluoride membranes (Millipore, Bedford, MA). After blocking with 5% non-fat dry milk, the membranes were incubated with primary antibodies overnight at 4°C, followed by washing and addition of a horseradish-conjugated secondary antibody for one hour. The protein bands were visualized with an Enhanced Chemiluminescence Plus Western Blotting Detection System (Amersham Biosciences, Piscataway, NJ).

### RT-PCR

Total RNA was extracted from cells using TRIZOL reagent (Gibco BRL, Rockville, MD). For reverse transcription-polymerase chain reaction (RT-PCR), 2 µg total RNA was reverse-transcribed with Superscript II (Gibco) in a 20-µl reaction solution. One tenth of the RT-PCR products was used for subsequent PCR analysis with 24–30 cycles of 95°C for 30 seconds, 58°C for 30 seconds, and 72°C for 30 seconds. The primers used were as follows: Human TACE sense 5′- ATTATTGGTGGTAGCAGATC-3′ and antisense 5′- GAGCCAACATAAGCTAATCC-3′. Human DR4 sense 5′-TGGCACACAGCAATGGGAACATAG-3′ and antisense 5′-GAAACACACCCTGTCCATGCACTT-3′; and human TIMP-3 sense 5′- GCAGATGAAGATGTACCGAG-3′ and antisense 5′- GTCTGTGGCATTGATGATGC-3′. Human GAPDH was used as a housekeeping gene for quantity normalization (sense 5′-AATCCCATCACCATCTTCCA-3′, antisense 5′-TGGACTCCACGACGTACTCA-3′).

### Transfection

TACE siRNA (5′-augaguuguaaccaggucagcuucc-3′ and 5′-ggaagcugaccugguuacaacucau-3′) and scrambled siRNA were purchased from Invitrogen. Three µg of each siRNA was transfected into cells seeded in 24-well plates by electroporation using a Human Nucleofector Kit (Amaxa Biosystems, Cologne, Germany).

### Cell viability assay

rhTRAIL was added into cultures. In cytotoxic analyses with R1-B12, R1-B12 was added into cultures followed by cross-linking with goat F(ab′)_2_ anti-human IgG Fc. After culturing for 2 days, viable cell numbers were counted by trypan blue dye exclusion assay as we previously described [29]. Cell viability was also determined by Cell Counting Kit-8 assay (DOJINDO, Kumamoto, Japan) according to the manufacture's instructions. Briefly, cells were seeded in 96-well plate and incubated with 2-(2-methoxy-4-nitrophenyl)-3-(4-nitrophenyl)-5-(2, 4-disulphophenyl)-2H-tetrazolium monosodium salt (WST-8) for 1–4 hours. The absorbance of each well was measured at 450 nm with a microtiter plate reader (Model 450 micro plate reader; Bio-Rad Laboratories, Hercules, CA). Apoptosis and cell death were evaluated by staining cells with an annexin V-FITC and PI labeling kit (MEBCYTO Apoptosis Kit; MBL,Nagano, Japan) according to the manufacture's instruction.

### Colony forming cell assays

CD138-negative bone marrow cells from a patient with MM were cultured in the absence or presence of R1-B12 or R1-B12 plus TAPI-0 for 14 days in semi-solid methylcellulose media (MethoCult H4034 Optimum, Stem Cell Technologies, Vancouver, BC, Canada) according to the manufacturer's instruction. Colonies representing the hematopoietic progenitor cells were counted under a scanning microscope and scored as burst-forming unit-erythroid (BFU-E) and colony-forming unit-granulocyte, macrophage (CFU-GM).

### Statistical analysis

Statistical significance was determined by one-way analysis of variance (ANOVA) with Scheffe's post hoc tests. The minimal level of significance was p = 0.05.

## Supporting Information

Figure S1 DR4 mRNA expression. RPMI8226, U266, OPC, TSPC-1, KMS12-BM, and MM.1S cells were cultured for 6, 12 and 48 hours in the presence or absence of TAPI-0 at 10 µM as indicated. DR4 mRNA expression was analysed by RT-PCR in RPMI8226 and U266 cells. GAPDH was used for quantity normalization.
(TIFF)
