# Supplementary figures and images for: Inhibition of TACE Activity Enhances the Susceptibility of Myeloma Cells to TRAIL
Source: PLoS One. 2012 Feb 28;7(2):e31594. doi: 10.1371/journal.pone.0031594 (PMC3289627; doi:10.1371/journal.pone.0031594)

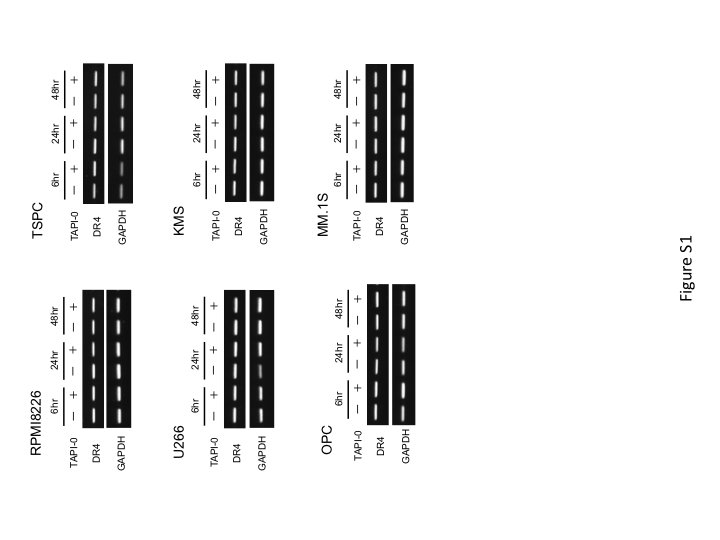

Supplement: Figure S1 — DR4 mRNA expression. RPMI8226, U266, OPC, TSPC-1, KMS12-BM, and MM.1S cells were cultured for 6, 12 and 48 hours in the presence or absence of TAPI-0 at 10 µM as indicated. DR4 mRNA expression was analysed by RT-PCR in RPMI8226 and U266 cells. GAPDH was used for quantity normalization. (TIFF) [file pone.0031594.s001.tiff]
